# Impact of merging commercial breeding lines on the genetic diversity of Landrace pigs

**DOI:** 10.1186/s12711-019-0502-6

**Published:** 2019-10-29

**Authors:** Ina Hulsegge, Mario Calus, Rita Hoving-Bolink, Marcos Lopes, Hendrik-Jan Megens, Kor Oldenbroek

**Affiliations:** 1 Animal Breeding and Genomics, Wageningen University & Research, P.O. Box 338, 6700 AH Wageningen, The Netherlands; 2 Centre for Genetic Resources, the Netherlands, Wageningen University & Research, P.O. Box 338, 6700 AH Wageningen, The Netherlands; 3 Topigs Norsvin Research Center, P.O. Box 43, 6640 AA Beuningen, The Netherlands; 4 Topigs Norsvin, Curitiba, PR 80420-210 Brazil

## Abstract

**Background:**

The pig breeding industry has undergone a large number of mergers in the past decades. Various commercial lines were merged or discontinued, which is expected to reduce the genetic diversity of the pig species. The objective of the current study was to investigate the genetic diversity of different former Dutch Landrace breeding lines and quantify their relationship with the current Dutch Landrace breed that originated from these lines.

**Results:**

Principal component analysis clearly divided the former Landrace lines into two main clusters, which are represented by Norwegian/Finnish Landrace lines and Dutch Landrace lines. Structure analysis revealed that each of the lines that are present in the Dutch Gene bank has a unique genetic identity. The current Dutch Landrace breed shows a high level of admixture and is closely related to the six former lines. The Dumeco N-line, which is conserved in the Dutch Gene bank, is poorly represented in the current Dutch Landrace. All seven lines (the six former and the current line) contribute almost equally to the genetic diversity of the Dutch Landrace breed. As expected, the current Dutch Landrace breed comprises only a small proportion of unique genetic diversity that was not present in the other lines. The genetic diversity level, as measured by Eding’s core set method, was equal to 0.89 for the current Dutch Landrace breed, whereas total genetic diversity across the seven lines, measured by the same method, was equal to 0.99.

**Conclusions:**

The current Dutch Landrace breed shows a high level of admixture and is closely related to the six former Dutch Landrace lines. Merging of commercial Landrace lines has reduced the genetic diversity of the Landrace population in the Netherlands, although a large proportion of the original variation is maintained. Thus, our recommendation is to conserve breeding lines in a gene bank before they are merged.

## Background

The pig is a major livestock species, which in 2016 accounted for 37% of the meat production worldwide [1]. The global pork production primarily relies on the use of a limited number of international commercial breeds, specifically Duroc, Large White, and Landrace. In the mid-twentieth century, a large number of breeding associations that operated regionally were responsible for pig breeding. Each of these breeding associations and breeding companies had their own breeding stock, which was usually based on the same limited number of commercial breeds, but often originated from national or regional, and therefore unique, populations.

Over the past decades, the commercial breeding industry has seen considerable business consolidation through mergers and take-overs, which have resulted in a limited number of remaining internationally operating breeding companies [2]. Consequently, the breeding lines owned by these companies have experienced a high degree of consolidation as well. Breeding lines that lost the competition in terms of performance and genetic gain were often discontinued but perhaps more often, breeding lines were merged ‘asymmetrically’, keeping the old breeding line’s name, but with extraneous influences.

The process of consolidation of breeding lines in domestic farm animals is most advanced in poultry, where both for broiler and laying chickens, the global market relies on just a handful of breeding lines/populations. Currently, the global poultry breeding market is primarily covered by just a few breeding companies, which has led to a loss of genetic diversity in these breeds [3]. Pig breeding shares similarities with poultry breeding in that it relies on a limited number of international breeds. Nevertheless, consolidation of pig breeding lines (and breeding companies, for that matter) has not yet progressed to the same extent. However, worldwide, genetic variation in pigs is threatened by the progressive marginalization of local breeds for the benefit of commercial breeds [4, 5]. The continued merging of the many distinct local populations of these commercial pig breeds and lines is expected to further increase the loss of genetic potential for pig production.

Traditional pig breeds and pure breeding lines are valued resources, not only for meat production, but also for cultural, historical, sociological, and environmental aspects. The underlying genetic variation may disappear, or may already have disappeared, from the global highly productive breeds that dominate modern intensive livestock production systems. Thus, the continued merging of breeding companies increases the concern of losing essential genetic variation [6].

The consolidation of breeding lines is often poorly documented, with public records usually limited or absent. Even for breeding lines listed by the FAO, which include data on their current status and vulnerability, information is often limited or outdated. A post hoc evaluation of loss of diversity in the aftermath of company mergers by genotyping is further hampered by the absence of reference samples from the pre-merger breeding lines. Here, we present a relatively well-documented case of the merging of a number of breeding associations that operated at the national level (the Netherlands) into an internationally operating breeding company (Topigs Norsvin). Although consolidation affected all breeding lines owned by the breeding companies, we will focus on one particular breed in this paper, i.e. the Dutch Landrace.

Our objective was to investigate the consequences of merging and discontinuing breeding populations on the genetic diversity of the Dutch Landrace breed over the past decades. To achieve this objective, we used genotype data of boars from the former Dutch Landrace breeding lines that have been conserved in the Dutch Gene bank to quantify their relationship with the current Dutch Landrace breed, and to estimate the loss (if any) of genetic diversity as a result of the merging of lines.

## Methods

### Description of the Landrace breed

**Fig. 1:**
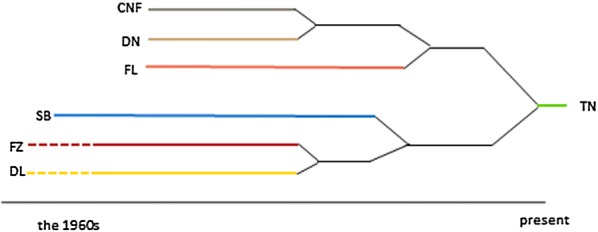
Timeline showing the consolidation of the Landrace breeds in the Netherlands since the 1960s (after [8]). *CNF* Cofok Norwegian and Finnish Landrace, *DL* Dumeco L-line, *DN* Dumeco N-line, *FL* Stamboek Finnish Landrace, *FZ* Fomeva Z1-line, *SB* Stamboek Dutch Landrace, *TN* Topigs Norsvin N-line

The Dutch Landrace breed originated from the original native Landrace pig, with infusions of the German Landrace and the Danish Landrace around 1900 [7]. By 1933, the Dutch Landrace was officially recognized as a Dutch native breed. By 1960, different breeding associations started selecting their own Dutch Landrace populations for their specific breeding goals. In the 1970s, Finnish and Norwegian Landrace pigs were imported into the Netherlands for use in crossbreeding programs [8]. During the 1990s, the Cofok, Dumeco, Fomeva, and Stamboek breeding associations, which together represent the majority of pig sales in the Netherlands, merged into a new internationally operating breeding organization called Topigs [9]. During this period, semen from breeding lines that were owned by the parent breeding organizations was deposited into the Dutch Gene bank (http://www.genebankdata.cgn.wur.nl/). This practice has been continued by Topigs (now called Topigs Norsvin) during the last two decades, resulting in a unique collection of material from breeding lines that were either discontinued or altered by merging lines. The timeline of consolidation of the different Landrace breeding lines in the Netherlands since 1960s is illustrated in Fig. 1 [8].

### Animals and genotypes

The Centre for Genetic Resources, the Netherlands (CGN) of Wageningen UR, i.e. the Dutch Gene bank, stores cryopreserved genetic material, primarily semen, from the former pig breeding associations in the Netherlands. From 1995 to 2003, CGN collected genetic material from six Landrace breeding lines of breeding associations that existed at that time. Merging of Dutch Landrace lines was in full progress and consequently the number of animals was already reduced. To select the group of boars, from the available animals, with minimal kinship and maximum diversity, optimal contributions were estimated using Gencont [10]. From 2011 to 2016, CGN has preserved genetic material from the current Dutch Landrace line (Topigs Norsvin N-line; hereafter referred to as “TN line”) in the Dutch Gene bank. Genotype data, provided by CGN and Topigs Norsvin, were available for 187 animals from six former Dutch Landrace lines (Dutch lines from Fomeva, Dumeco and Stamboek, and Dutch Norwegian/Finnish lines from Cofok, Dumeco and Stamboek) and the current TN line (Table 1).

**Table 1:** Number of genotyped animals in six former and the current Dutch Landrace line (TN line)

Line	Abbreviation	Origin of the lines^a^	Semen collection year	Number of animals
Cofok Norwegian and Finnish Landrace	CNF	FN	2000–2002	46
Dumeco L-line	DL	NL	1998–2002	49
Dumeco N-line	DN	FN	1998–2002	24
Stamboek Finnish Landrace	FL	FN	2002	11
Fomeva Z1-line	FZ	NL	2000	11
Stamboek Dutch Landrace	SB	NL	2002–2003	12
Topigs Norsvin N-line	TN	TN	2011–2016	34

^a^Origin of the lines: FN: Finnish/Norwegian; NL: Dutch; TN: current line

The 187 animals were genotyped using the PorcineSNP80 BeadChip (Illumina Inc., San Diego, CA, USA). All samples had a genotype call rate higher than 90%. For quality control, SNPs with a GenCall score lower than 0.20, a minor allele frequency lower than 0.02 and a per SNP genotype call rate less than 100% were removed from further analyses, the latter because some of the subsequent analyses cannot deal with missing genotypes. Imputing missing genotypes was not appropriate for this dataset, since it requires more animals for each of the lines involved to be genotyped. In addition, applying a call rate threshold of 100% left a sufficient number of SNPs in the dataset for subsequent analyses. The final dataset included 42,655 SNPs with calls for all 187 animals.

### Population structure

To examine relatedness between the Landrace lines, a principal component analysis (PCA) was performed using the prcomp function in R [11]. To identify subpopulations (clusters), genotypes of all individual animals were analysed by the model-based clustering algorithm implemented in the software Structure (version 2.3.4) [12, 13]. Subpopulation numbers (K) ranging from 2 to 7 were evaluated by repeating each analysis 10 times. A burn-in of 10,000 iterations and subsequent 50,000 iterations of the Markov chain Monte Carlo were applied, with all other program parameters set to their default values. The most likely number of subpopulations was inferred with the ΔK method of Evanno [14], implemented in the R package pophelper (version 2.2.3) [15]. The program CLUMPP [16] implemented in pophelper was used to align the 10 independent runs for each K. Pophelper was also used to plot results for K = 2 to 7. The Structure analysis was performed a second time by applying the “Use Population Information” setting, such that individuals of the TN line (POPFLAG = 0) were assigned to clusters that were defined by the allele frequencies of the other lines (POPFLAG = 1). A neighbour-joining tree [17] was computed based on the resulting distance matrix using the R package APE (version 4.1) [18]. Genetic divergence between each pair of Landrace lines was quantified by calculating pairwise *F*st, as defined by Weir and Cockerham [19], using the R-package ‘hierfstat’ (version 0.04–22) [20].

### Genetic diversity

The contribution of breeds to genetic diversity was analysed using the marker-estimated kinships and the core set method of Eding et al. [21]. In this method, kinship coefficients are estimated based on SNP genotypes, and the genetic diversity within a breed is estimated as one minus the average kinship coefficient in that breed. The average kinship coefficient was also estimated across breeds to determine the genetic diversity of the whole set. The total genetic diversity of a set depends on the contribution of each breed to the total set. If all breeds contribute equally, the total genetic diversity is equal to one minus the average within- and across-breed kinship coefficients. Otherwise, the kinship coefficients of each breed have to be weighted by their contribution, as:

$${\text{g}}_{\text{div}} = 1 - {\mathbf{c}}'{\mathbf{Mc}},$$

where ${\mathbf{c}}$ is a vector of the $n$ (number of breeds) contributions of each breed (summing to 1) and ${\mathbf{M}}$ is a $n \times n$ matrix with within- and across-breed kinship coefficients. Thus, if a relatively uniform breed contributes more to the total set, the genetic diversity of the total set will be lower than when a relatively diverse breed contributes.

In the core set method of Eding et al. [21], the contribution of each breed that maximizes the genetic diversity is estimated as:

$${\mathbf{c}}_{{{\mathbf{max}}}} = \frac{{{\mathbf{M}}^{ - 1} {\mathbf{1}}_{n} }}{{{\mathbf{1}}_{n}  \varvec{'} {\mathbf{M}}^{ - 1} {\mathbf{1}}_{n} }},$$

where ${\mathbf{c}}_{{{\mathbf{max}}}}$ is the vector of contributions that maximizes the diversity in the total set, ${\mathbf{1}}_{n}$ is a vector of $n$ 1s, and ${\mathbf{M}}$ is the $n \times n$ matrix with the average within- and between-breed kinships. Then, the total diversity in the set is estimated as:

$$Div_{set} = 1 - {\mathbf{c}}_{{{\mathbf{max}}}} '{\mathbf{Mc}}_{{{\mathbf{max}}}} = \frac{1}{{{\mathbf{1}}_{n} \varvec{'}{\mathbf{M}}^{ - 1} {\mathbf{1}}_{n} }}.$$

Thus, the contribution of each breed to this core set depends on both the between- and within-breed components of genetic diversity. However, this contribution is not the only one that determines the relative importance of a breed to total genetic diversity. A breed that only contributes a small amount to the core set (e.g. when their within-breed kinship is high) can, nevertheless, increase the total genetic diversity considerably, e.g., when its across-breed kinships are low. Therefore, the average kinship coefficient of the core set when the breed is included is compared to the average kinship coefficient of the core set when the breed is excluded [21].

The required kinship coefficients were obtained by first computing the genomic relationship matrix (${\mathbf{G}}$) according to Yang et al. [22], using the software Calc_grm [23]. Using ${\mathbf{G}}$, average within- and between-breed kinship coefficients were computed across all pairwise relationships within and between breeds, including self-kinship coefficients.

### Identification of selection signatures by using *F*st

Selection signatures were detected for each pairwise comparison between the current TN and the six former lines, by using the *F*st-outlier approach implemented in the BayeScan software (version 2.1), using default settings [24]. SNPs with a q-value lower than 0.05 were considered as outliers, which indicate regions potentially under selection. Genes that are located within 10 kb (5 kb downstream/upstream) of the SNP outliers were identified as candidate genes, based on the Ensembl annotation of Sscrofa10.2 (https://may2017.archive.ensembl.org/Sus_scrofa/Info/Index). The candidate genes were characterized using the PANTHER Classification System version 14.1 (http://geneontology.org/) [25], in particular, with the GO-Slim Biological Process annotation dataset. Overrepresentation analysis of GO-Slim Biological Process terms was also done using PANTHER; GO terms with a p ≤ 0.05 after Bonferroni correction were deemed significant. Compared to using the entire GO term database, GO-Slim uses a limited set of GO terms to provide a more general list of functions that map to genes.

## Results

### Population structure

**Fig. 2:**
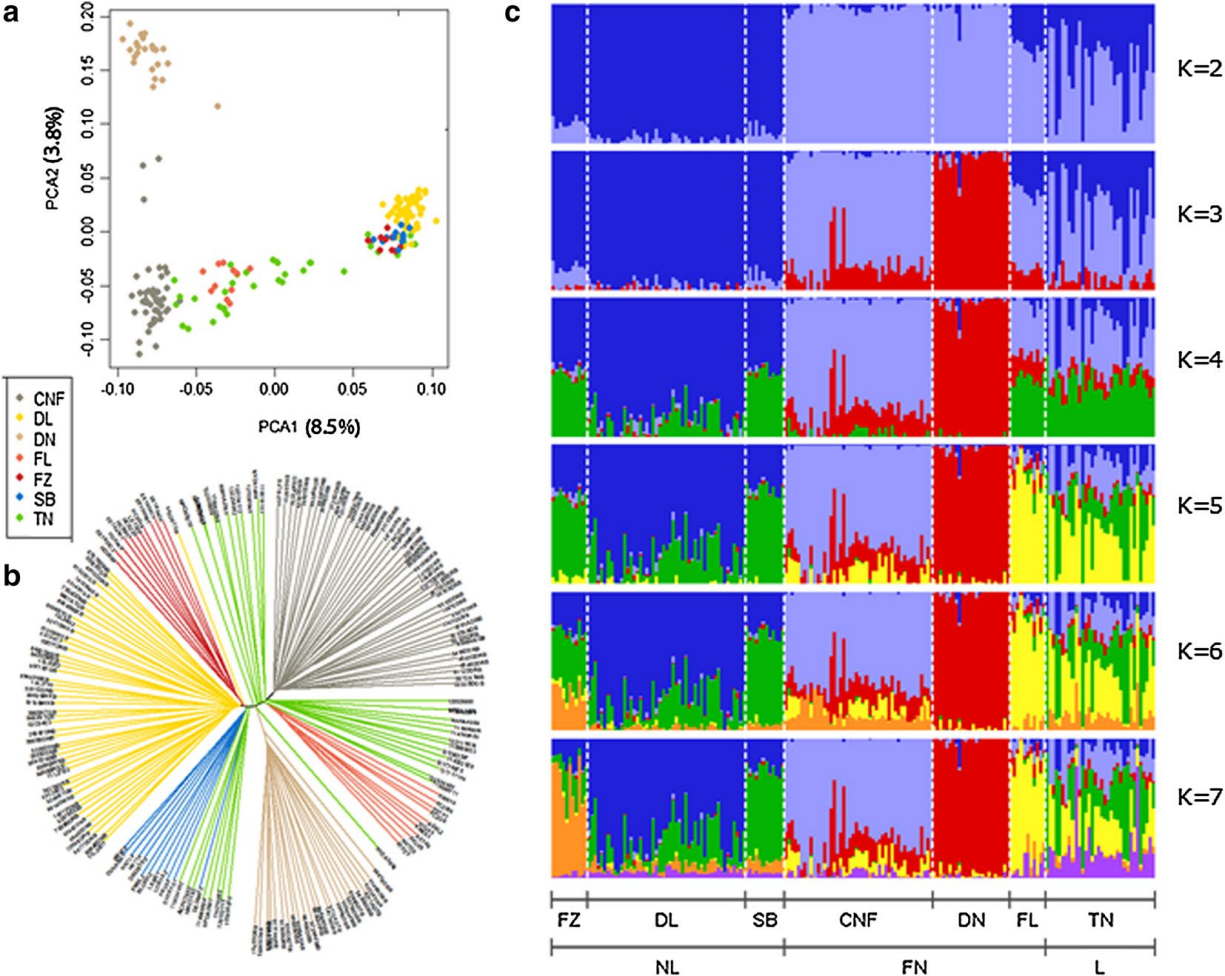
Population structure and relationships of Landrace breeding lines in the Netherlands. **a** Principal component (PC) analysis, PC 1 against PC 2. **b** Neighbor-joining tree of the relationships between the seven lines. **c** Proportion of ancestry for each individual assuming different numbers of ancestral populations (K = 2 to 7). Colors of each vertical line represent the estimated proportion of an animal’s genome that is assigned to a source population

The current Dutch Landrace (TN: Topigs Norsvin N-line) is the result of the consolidation of six former Landrace lines that existed from the 1960s until early 2000 (CNF: Cofok Norwegian and Finnish landrace, DL: Dumeco L-line, DN: Dumeco N-line, FL: Stamboek Finnish Landrace, FZ: Fomeva Z1-line and SB: Stamboek Dutch Landrace). The PCA clearly indicates a division of the seven Landrace lines into two main clusters; on the one hand, the former Norwegian/Finnish Landrace lines (CNF, DN and FL lines), which were introduced in the Netherlands between 1970 and 1980, and, on the other hand, the former Dutch Landrace lines (DL, FZ and SB) (Fig. 2a). Clearly, the current commercial Dutch Landrace line (TN) is a mixture of the former breeding lines, since the old breeding lines included the extremes of the first principal component (PC1). The widespread distribution of the animals along PC1 for the current TN line shows that the contribution of the Dutch and Norwegian/Finnish lines to the current line differs between pigs. The second principal component (PC2) distinguished the DN line from the other six lines.

A unique genetic identity was identified for each of the six former Landrace lines based on the cluster analysis using the Structure software (Fig. 2c). At K = 2, the two ancestries clearly reflected Dutch Norwegian/Finnish versus Dutch Landrace origins. At K = 3, DN was separated from CNF and FL (representative of Dutch Norwegian/Finnish Landrace). Based on ΔK, the most likely number of genetic groups (clusters) was equal to 5. While all parent lines appeared to be well separated at K = 5 (with the exception of FZ and SB), TN is clearly an admixed population with substantial contributions from the former breeding lines CNF, FL, and FZ/SB. At K = 5, the average proportion of membership of the founder breeds to TN was 0.205, 0.043, 0.350, 0.290, and 0.112 for CNF (cluster 1), DN (cluster 2), FL (cluster 3), FZ/SB (cluster 4), and DL (cluster 5), respectively. Results of the Structure analysis with no prior population information (POPFLAG = 0 for TN line) is shown in Figure S1 (See Additional file 1: Figure S1), and confirmed the results of the PCA, i.e. that the contributions from the former lines differed between individuals. A neighbor-joining tree separated the breeding lines from each other in separate clades, except for the current TN line (Fig. 2b).

Genetic differentiation among the Landrace lines was low to moderate, as indicated by the pairwise *F*st values that ranged from 0.02 to 0.10 (Table 2). The genetic differentiation of the current TN breeding line from the six former lines was low, which indicates that the current breeding line is closely related to the former breeding lines.

**Table 2:** Estimated pairwise *F*st as a measure of genetic differentiation (below the diagonal) and average genomic kinship (above the diagonal) between the Landrace breeding lines

	CNF	DL	DN	FL	FZ	SB	TN
CNF	–	− 0.072	0.044	0.008	− 0.092	− 0.091	0.013
DL	0.066	–	− 0.070	− 0.072	0.025	0.048	− 0.019
DN	0.051	0.077	–	− 0.018	− 0.109	− 0.105	− 0.033
FL	0.036	0.044	0.074	–	− 0.074	− 0.087	0.010
FZ	0.055	0.030	0.098	0.088	–	0.025	− 0.034
SB	0.054	0.024	0.094	0.085	0.0588	–	0.039
TN	0.032	0.035	0.061	0.029	0.0412	0.0310	–

### Genetic diversity

The average kinship coefficients between and within the Landrace lines are in Tables 2 and 3. As expected, within-line kinship coefficients were higher (Table 3; ranging from 0.051 to 0.249) than the between-line kinship coefficients (Table 2; ranging from − 0.092 to 0.074). The higher negative between-line kinship coefficients between the former Dutch Norwegian/Finnish and the Dutch breeding lines indicates that the distance between these lines was greater than between individuals within the lines. The within-line kinship coefficient was lowest (0.051) for the current TN.

**Table 3:** Average genomic kinship coefficient ($\overline{\varvec{f}}$) within lines and the contribution of lines to a core set in which the diversity is maximized (= $\overline{\varvec{f}}$ minimised)

Line	$\overline{\varvec{f}}$	Contribution (%)	Unique diversity
CNF	0.170	15.74	0.005
DN	0.249	17.79	0.008
FL	0.158	14.70	0.007
DL	0.143	12.45	0.007
FN	0.186	13.28	0.004
SB	0.121	10.84	0.004
TN	0.051	15.18	0.003
Core set	0.007		–

Unique genetic diversity is measured as the increase in $f$ when the core set is formed without a contribution of that breed

The contribution of each line (in %) to the genetic diversity in the overall Landrace population is in Table 3. All lines contributed to the diversity of the core set. The largest contribution to the total genetic diversity of the Landrace breed was observed for DN (17.79%), whereas it was smallest for SB (10.84%). Each line had a certain proportion of unique genetic diversity. The total genetic diversity of the Landrace breeding lines, estimated by Eding’s core set method, was 0.993, and that of the six former breeding lines was 0.990, while the genetic diversity of TN was 0.894.

### Identification of selection signatures using *F*st

**Fig. 3:**
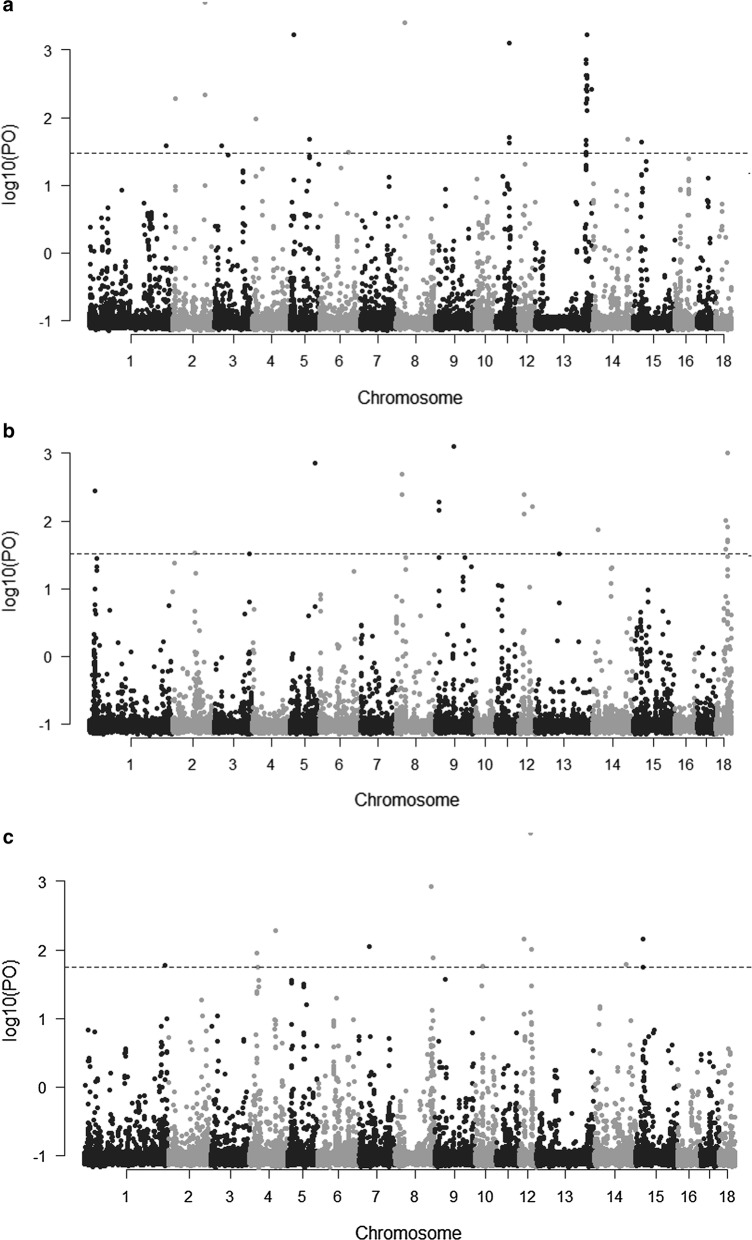

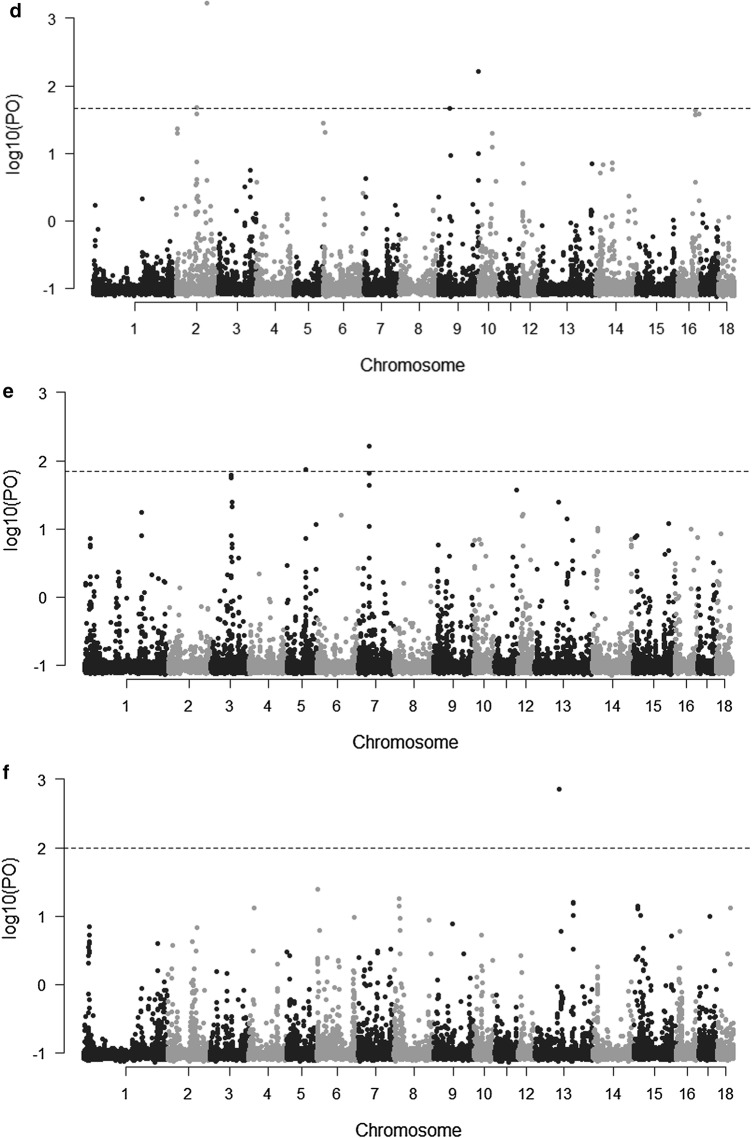
Genome-wide distribution of log10 Bayes factor values in the pairwise comparison between the current TN and the six former lines. **a** CNF versus TN, **b** DL versus TN, **c** DN versus TN, **d** FL versus TN, **e** FZ versus TN and **f** SB versus TN. The threshold for significance of signatures of selection is denoted with a line (q-value ≤ 0.05)

As breeding lines are merged, selection continues, although in some cases the breeding goal may be different in the consolidated line compared to the parent lines. SNP genotypes were used to estimate allele frequency differentiation (measured as *F*st) in pairwise comparisons between the current TN and the six former lines. Outlier (high allele frequency differentiation) SNPs are an indication of regions that are potentially under selection. The 10log Bayes factor values for each SNP are shown in Fig. 3. The number of loci with statistically significant patterns of divergent genetic differentiation (q-value ≤ 0.05), which were identified by pairwise comparisons, revealed that CNF and TN had the largest number (93) of outlier SNPs (Table 4). The outlier SNPs were located close to or within 20 candidate genes. Among these outlier SNPs, 29% (n = 27) were located almost at the end of chromosome 13 (SSC13: 191,713,636–196,766,412). Almost all of these 27 outliers are intergenic variants, which lie in-between genes (see Additional file 2: Table S1). Additional file 2: Table S1 lists the outlier SNPs, candidate genes, and their respective assigned GO-slim terms (Biological Processes). Fifty-three SNPs were identified as loci that were under diversifying selection between the DL and TN lines, and these corresponded to 20 candidate genes. Seven outlier SNPs were located within small nucleolar RNAs (snoRNAs). Pairwise comparison between DN and TN revealed 46 SNP outliers (q-value ≤ 0.05) with 13 candidate genes. Pairwise comparisons of TN with each of the other four lines revealed 46 significant SNPs (q-value ≤ 0.05) between DN and TN, 18 between FL and TN, 21 between FZ and TN, and 7 between SB and TN. No candidate genes were found for the comparison between SB and TN. GO annotation of the candidate genes showed that most genes were linked to biological processes associated with cellular processes, metabolic processes, and intracellular transport (Table 4) and (see Additional file 2: Table S1). However, no significant over-representation was observed for any biological process.

**Table 4:** Number of outlier SNPs detected (q-value ≤ 0.05) by BayeScan and their respective candidate genes within 5 kb up- or downstream

Pairwise comparison of lines	Number of outlier SNPs	Candidate genes	General term GO BP
CNF–TN	93	*CDC6*, *CIB4*, *CLEC1A*, *CLEC7A*, *ENSSSCG00000000959*, *ENSSSCG00000007221*, *ENSSSCG00000008799*, *ENSSSCG00000012012*, *ENSSSCG00000020566*, *ENSSSCG00000025389*, *ENSSSCG00000027643*, *ENSSSCG00000027841*, *ENSSSCG00000028250*, *GALM*, *GDE1*, *LAPTM5*, *LRRK2*, *RAB39A*, *SLC35F2*, *TCN1*	Cell cell signaling/immune, cell communication, cell cycle, intracellular transport, metabolic process, skeletal muscle function and regeneration, system process, transmembrane transport
DL–TN	53	*ABRACL*, *BECN1*, *CCDC6*, *ENSSSCG00000010218*, *GARS*, *KIAA0513*, *MINDY4*, *NOL10*, *REPS1*, *SPNS2*, *SPNS3*, *UST*, *WNK4*	(Cell) development/differentiation, cell communication, cellular processes, gene expression, intracellular transport, metabolic process
DN–TN	46	*CACNG3*, *CD48, CFAP45*, *DDX42*, *ENSSSCG00000024706*, *ENSSSCG00000026756*, *ENSSSCG00000027460*, *GLO1*, *GOT1, KSR1*, *PKHD1L1*, *ssc*-*mir*-*4331*, *TSPAN11*	Cell communication, cellular processes, immune, intracellular transport, metabolic process
FL–TN	18	*EHBP1L1*, *KCNK7*, *MPP7*, *VAT1L*, *ZNF354C*	Intracellular transport, metabolic process
FZ–TN	21	*FZD2*, *IL17REL*, *PLEKHM1*, *WDR92*	Cellular process, developmental processes, intracellular transport
SB–TN	7	–	

## Discussion

In this study, we investigated the consequences for genetic diversity of the merging of lines within a breed, with the individual lines being discontinued thereafter. The data were derived from samples of boars that are included in the Dutch Gene bank collection, which led us to assume that these would encompass the genetic variation present in the modern breeding population [26]. Sample size of some Dutch Landrace lines used in this study were relatively small, due to limited availability of samples, and differed between lines, ranging from 11 samples for the lines FL and FZ lines to 49 for the DL line. Small sample size can lead to incorrect estimates of allele frequencies [27] and a proportion of genetic diversity present in the lines may remain undetected. Nevertheless, the results showed that the sampled animals formed genetic clusters that corresponded to their line designations (Fig. 1). The results also showed that, genetically, the current commercial Dutch Landrace line (TN) is a mixture of the six former Landrace lines in the Netherlands. In general, the results reported here are in good agreement with the known history of the different Landrace lines examined [8, 9].

### Genetic diversity

Genetic differentiation between the lines (pairwise *F*st) was moderate to low. Wilkinson et al. reported a mean *F*st value of 0.156 between three British Landrace lines [28]. For wild pigs sampled across different locations of the state of Florida (USA), pairwise *F*st values ranged from 0.020 to 0.256 [29]. The *F*st values (0.02 to 0.10) found in our study are at the lower end of this range. According to Willing et al. [30], *F*st can be accurately calculated based on small sample sizes (as small as n =  4 to 6) if the number of markers examined is large, i.e. larger than 1000.

The results reported here show that the merging of commercial Landrace lines has reduced the genetic diversity of the Landrace population in the Netherlands. For poultry, Besbes et al. [31] also reported that the merging of lines leads to a decrease in genetic diversity of the available gene pool. However, our results also showed that, after merging, a large proportion of the genetic variability was maintained, and that all former lines showed a lower genetic diversity than the current TN. This indicates that merging lines is a better strategy for maintaining genetic diversity than just continuing with one line and discontinuing the other lines.

In this study, the total genetic diversity of the Landrace lines was estimated using the optimal contribution strategy. The optimal contributions of breeding lines were derived such that the average kinship coefficient in the core set was minimal, and thus the genetic diversity was maximal. Because breeding programs compete for market share, they select their lines intensively. Due to the breeding strategies that were followed over time, the actual genetic contributions of the different parent lines to the current Landrace line differed from the optimal contributions, indicating that part of the genetic diversity was lost. In addition, the DN line was poorly represented in the current Dutch Landrace. These observations support the recommendation that all breeding lines should be conserved before merging and discontinuing them.

### Identification of selection signatures using *F*st

Commercial pig breeds have been subject to intense artificial selection for production traits. Functional analysis of regions under positive selection in pig breeds has identified genes that are involved in the development of the nervous system and of muscle, and in growth, pigmentation, metabolism, visual/odour perception, immune and inflammatory responses, and reproduction [32]. Functional annotation analyses of the candidate genes in our study are shown in Table 4. For the interpretation of our results, it should be noted that we used the Ensembl annotation of Sscrofa10.2 and not the latest version Sscrofa11.1 [33]. Furthermore, a small sample size can lead to poor population structure estimates, which affects the ability to differentiate between loci that were under selection and neutral population structure [34]. However, in our study at least 11 animals per line were used, in line with a previous study that suggested that detecting regions under selection with *F*st methods requires at least 10 samples [30].

We detected no over-representation of any GO biological process among the candidate genes in our study. It should be noted that most traits that are under selection in pigs are complex traits that are regulated by many genes [35]. We identified a number of candidate genes that were located within 10 kb (5 kb downstream/upstream) of the SNP outliers, most of them being associated with cellular processes, metabolic processes and intracellular transport (Table 4) and (see Additional file 2: Table S1). The candidate genes that were found in the comparison between the CNF and FN lines are involved in fertility (*LAPTM5* [36]; *CIB4* (sheep) [37]), the immune system (*RAB39A* [38]), and intramuscular fat content (*ENSSSCG00000012012* [39]). In the comparison between the DL and TN lines, we identified *BECN1*, which is a muscle-related gene [40, 41], *GARS* and *NOL10*, which are associated with meat quality [42, 43], and *KIAA0513*, which is associated with the male reproduction trait “Seminiferous tubule diameter” [44]. In the comparison between the DN and TN lines, we detected several candidate genes: *GLO1*, which is assumed to be involved in fatness [45], is important for nutrition energy intake and obesity [46], and is connected with pig birth weight variability [47]; *GOT1* and *PKHD1L1*, which have been reported as candidate genes for intramuscular fat content [48] and variation in pH of meat [49], respectively; and *TSPAN11*, which was associated with metabolic body weight in a study on Holstein dairy cows [50]. In the comparison between the FZ and TN lines, we found the candidate gene *WDR92*, which is associated with total fat in Duroc and Yorkshire F2 intercrosses [51]. It should be kept in mind that, although these associated SNPs and respective genes may be involved in certain biological processes related to selection events, further experimentation needs to be performed to verify these associations.

As shown by our results, differences can be pronounced even between populations that have common origins, which stresses the value of gene banks to record and preserve variation that is lost in the process of merging, even over short periods of time.

### Consolidation

The breeding industry has undergone a strong consolidation process in the past decades and this will likely continue [52–54]. Economic reality forces breeding companies to discard breeding lines that are not of immediate value for product formulation or do not have potential to be used in the near future. Inevitably, maintaining genetic diversity in breeds and breeding lines has a cost, while the benefits are not immediately translated into profit. However, the consequences of losing genetic diversity are generally acknowledged; maintaining it is essential to provide future opportunities of selection for changing markets, consumer preferences, products etc., to allow sustained genetic improvement, to develop alternatives to intensive management, to decrease disease incidence and increase health, and to anticipate future changes in climate [52, 55–57].

## Conclusions

The current Dutch Landrace (TN line) shows a high level of admixture and is closely related to the six former Dutch Landrace lines. However, the merging of commercial Landrace lines has reduced the genetic diversity of the Landrace population in the Netherlands, and the DN line is poorly represented in the current Dutch Landrace. Thus, it is recommended to conserve selection lines in a gene bank before merging. Our findings also showed that the merging of lines results in a large proportion of the original variability being maintained.

## Supplementary information

**Additional file 1: Figure S1.** Inferred ancestry of the 34 TN line individuals. Each bar is an individual.

**Additional file 2: Table S1.** Outlier SNPs identified (q-value ≤ 0.05) by BayeScan in the pairwise comparison between the current TN and the six former lines. SNP ID, SNP identifier; Chromosome, chromosome number; Position, position on the chromosome; prob, posterior probabilities; log10(PO), the logarithm to 10 of Posterior Odds; qval, q-value the threshold for significance of signatures of selection; alpha, a locus-specific component indicating the strength and direction of selection, a positive value of alpha suggests diversifying selection, whereas negative values suggest balancing or purifying selection; fst, fixation index; RS ID, accession number of the SNP; Gene Name (5 kb upstream/downstream), name of a gene that is located within 10 kb (5 kb downstream/upstream) of the SNP outlier; GO BP (GO-slim), Gene Ontology slim biological process term; General Term GO BP, general Gene Ontology biological process term.

## Data Availability

The data that support the findings of this study are available from the Centre for Genetic Resources, the Netherlands and Topigs Norsvin but restrictions apply to the availability of these data, which were used under license for the current study, and thus are not publicly available. However, data are available from the authors upon reasonable request and with permission from the Centre for Genetic Resources, the Netherlands and Topigs Norsvin.
