# Supplementary figures and images for: Impact of merging commercial breeding lines on the genetic diversity of Landrace pigs
Source: Genet Sel Evol. 2019 Oct 29;51:60. doi: 10.1186/s12711-019-0502-6 (PMC6819590; doi:10.1186/s12711-019-0502-6)

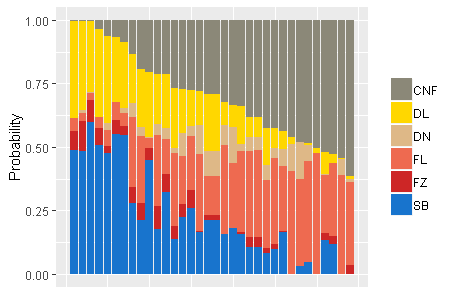

Supplement: Supplementary file 1 — Additional file 1: Figure S1. Inferred ancestry of the 34 TN line individuals. Each bar is an individual. [file 12711_2019_502_MOESM1_ESM.tiff]
